# Mechanical overload decreases tenogenic differentiation compared to physiological load in bioartificial tendons

**DOI:** 10.1186/s13036-022-00283-y

**Published:** 2022-03-03

**Authors:** Stefan Pentzold, Britt Wildemann

**Affiliations:** Experimental Trauma Surgery, Department of Trauma, Hand and Reconstructive Surgery, Jena University Hospital, Friedrich Schiller University Jena, Am Klinikum 1, 07747 Jena, Germany

**Keywords:** Tenogenesis, Gene expression, Cytoskeleton, Mechanical strain, Inflammation, C3H10T1/2

## Abstract

**Background:**

Tenocytes as specialised fibroblasts and inherent cells of tendons require mechanical load for their homeostasis. However, how mechanical overload compared to physiological load impacts on the tenogenic differentiation potential of fibroblasts is largely unknown.

**Methods:**

Three-dimensional bioartificial tendons (BATs) seeded with murine fibroblasts (cell line C3H10T1/2) were subjected to uniaxial sinusoidal elongation at either overload conditions (0–16%, Ø 8%) or physiological load (0–8%, Ø 4%). This regime was applied for 2 h a day at 0.1 Hz for 7 days. Controls were unloaded, but under static tension.

**Results:**

Cell survival did not differ among overload, physiological load and control BATs. However, gene expression of tenogenic and extra-cellular matrix markers (*Scx*, *Mkx*, *Tnmd*, *Col1a1* and *Col3a1)* was significantly decreased in overload *versus* physiological load and controls, respectively. In contrast, *Mmp3* was significantly increased at overload compared to physiological load, and significantly decreased under physiological load compared to controls. *Mkx* and *Tnmd* were significantly increased in BATs subjected to physiological load compared to controls. Proinflammatory interleukin-6 showed increased protein levels comparing load (both over and physiological) *versus* unloaded controls. Alignment of the cytoskeleton in strain direction was decreased in overload compared to physiological load, while other parameters such as nuclear area, roundness or cell density were less affected.

**Conclusions:**

Mechanical overload decreases tenogenic differentiation and increases ECM remodelling/inflammation in 3D-stimulated fibroblasts, whereas physiological load may induce opposite effects.

**Supplementary Information:**

The online version contains supplementary material available at 10.1186/s13036-022-00283-y.

## Introduction

Tendons connect force-generating muscles to bones to enable motion of humans and animals. Therefore, tendons and their inherent cells such as tenocytes as specialised fibroblasts are naturally under mechanical load. This force is essential for the homeostasis of tenocytes and whole tendons, but only a certain range of loading enhances net matrix production and thus tissue repair [1]. Whereas physiological relevant load in terms of elongation has important implications for the development and repair of tendons, overload and/or overuse can cause tendon impairments and injuries [2, 3]. Acute and chronic tendon injuries like tendinopathies occur frequently not only among older, less active and overweight persons, but also young, active persons and athletes are affected [4]. Moreover, tendons have limited self-healing capacity and current treatments (e.g. ultrasound, corticosteroids injection, surgery) have many limitations [1]. Therefore, distinguishing overload as a pathological condition from physiological and thus beneficial load would optimise loading regimes which could finally improve existing rehabilitation programs and help to engineer improved tendon constructs.

Strategies to induce the tenogenic differentiation potential of cells involves the application of mechanical strain mainly from 1 to 12% [1], which is imposed on individual cells or whole tissues, among other treatments such as growth factor supply and various scaffold surface structures [5–7]. However, conditions for overload or physiological load often vary among cell types and loading systems [8]. Similarly, the precise level (magnitude, frequency, duration) of stimulation required for normal tendon homeostasis *in vivo* often remains unknown [9]. For example, 9% cyclic strain (at 0.25 Hz) can cause damage to cells compared to 6% and 3% strain using Achilles tendons cultured *ex vivo* in growth medium [3]. Mechanical overloading at 12% strain of human tenocytes was shown to result in disruption of the cytoskeleton in a time-dependent manner [10]. Similar to overload unloading leads to increased cell apoptosis and collagen disorientation [3, 11].

Engineering of bioartificial tendons (BATs) such as by embedding tenocytes or fibroblasts cells in a three-dimensional (3D) matrix of type 1 collagen and simultaneous application of mechanical load increases expression of tendon-related genes such as *Scleraxis* (*Scx*) and *Type 1 collagen alpha 1* (*Col1a1*) with increasing strain [12]. The transcription factor *Scx* induces *Tenomodulin* (*Tnmd*), another important tendon-related marker mainly indicating differentiated and mature tenocytes [13]. *Tnmd* expression in mice Achilles tenocytes can be induced by 5% strain under 2D cultivation [14]. The transcription factor Mohawk (*Mkx*) is a further important tendon-related marker indicating tendon development [15, 16]. *Type 3 collagen alpha 1* (*Col3a1*) is a minor component in tendons in comparison to *Col1a1*, but its ratio may change under load [1] or during tendinopathy [17]. Matrix metalloproteinases (e.g. *Mmp3*) are involved in extra cellular matrix (ECM) remodelling and may even have pro-inflammatory roles in tenocytes [18], which may be pronounced under inflammatory conditions [19] or overload conditions. Finally, a typical cytokine in tendons is interleukin IL-6 that is strongly increased under pro-inflammatory stimulation using 3D tendon-like constructs *in vitro* [19].

In this study, we use C3H10T1/2 cells, a murine cell line of mesenchymal stem cells that show a fibroblastic phenotype and are often used for engineering bioartificial tendons or similar 3D constructs [12, 20–22]. C3H10T1/2 cells possess the ability to differentiate into cell lineages related to the musculoskeletal system (e.g. chondrocytes, osteocytes, tenocytes) under certain mechanical cues rendering them an ideal system to study tendon differentiation under load [22]. We analyse tendon, ECM- and inflammation markers together with histological analyses to reveal if cyclic elongation of fibroblasts-laden BATs at overload (0–16%) or physiological conditions (0–8%) inhibits or favours tenogenic differentiation or inflammation, respectively.

## Materials and methods

### Cell culture and BAT engineering

Cells of the murine fibroblast cell line C3H10T1/2 (Clone 8, ATCC® CCL226™, American Type Culture Collection, Manassas, VA) were propagated in DMEM/F12 culture medium (P04-41150, PAN-Biotech, Aidenbach, Germany) supplemented with 10% FBS superior (fetal bovine serum, 0708G, Biochrom AG, Berlin, Germany) and 1% penicillin-streptomycin (15140, Gibco™, Fisher Scientific GmbH, Schwerte, Germany) in standard cell culture T-flasks by incubation at 37 °C and 5% CO_2_; at 80% confluence cells were passaged. Cells were trypsinized using trypsin-EDTA 0.05% (25300054, Gibco™) for 5 min at 37 °C, pelleted at 200 g for 5 min and seeded at a concentration of 1.5 × 10^5^ per BAT. Therefore, cells were resuspended in 90 µl PureCol® EZ gel type I collagen solution (5074-G, Advanced BioMatrix, San Diego, CA, USA), 45 µl culture medium and 15 µl FBS and pipetted into each well of a linear type I collagen coated TissueTrain® culture plate (TT-5001 C, FlexCell International, Hillsborough, NC, USA) in which a trough was applied by underpressure using the FX-6000T™ Tension System (FlexCell International). The construct was allowed to set for 2 h, then covered with 3 ml of culture medium and incubated for 7 d to form tendon-like structures; medium was changed every 48 h. Each BAT construct was considered as an individual sample.

### Mechanical loading regime

**Fig. 1:**
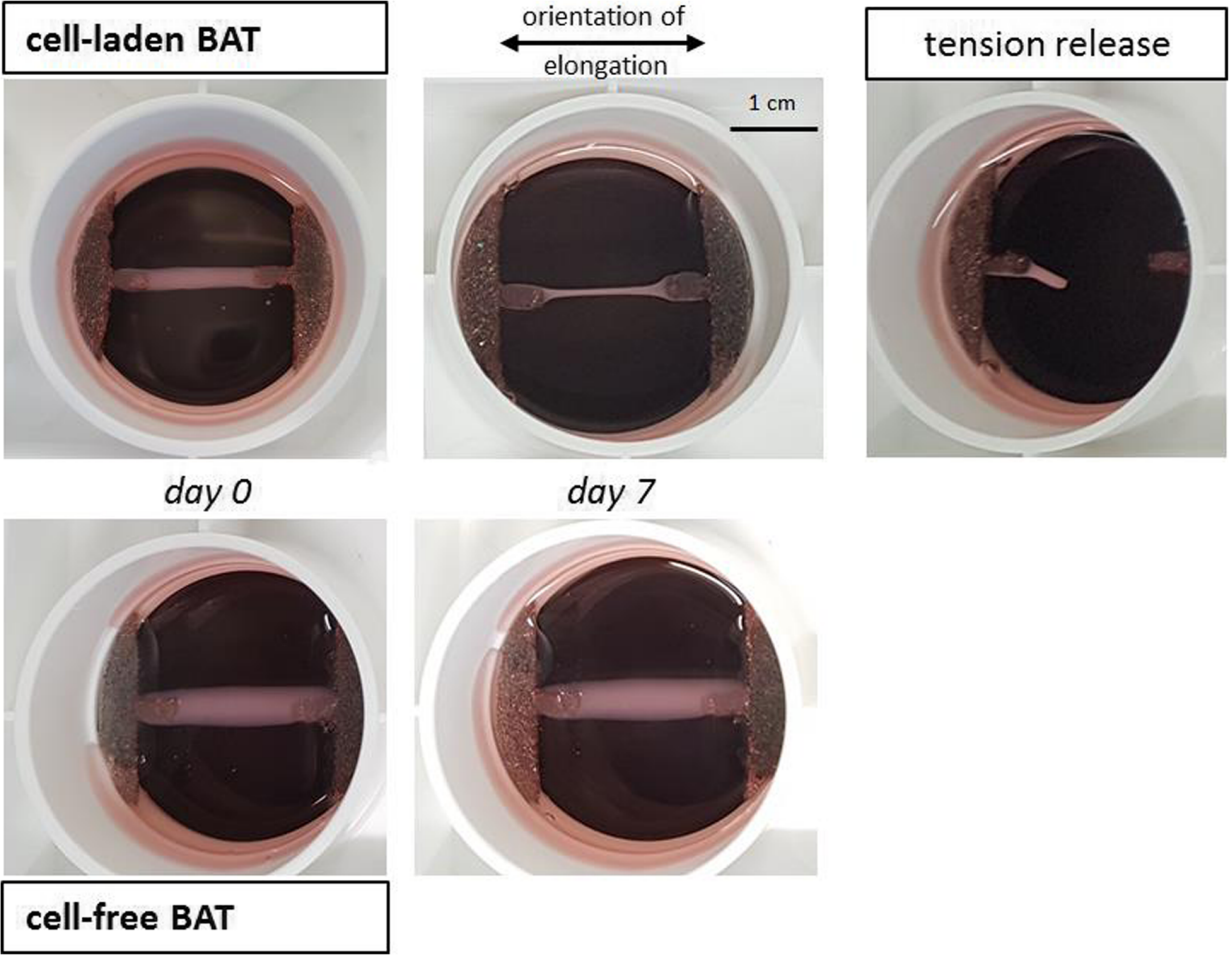
Appearance of bioartificial tendons (BATs) two hours after seeding C3H10T1/2 murine fibroblasts in a collagen type I matrix (day 0), or after maturation and prior to mechanical elongation (day 7). Whereas cell-laden BATs become thinner over time due to cell-based matrix remodelling and thus appear tendon-like, cell-free BATs do not change their appearance over time and stay loose and friable. Moreover, when cell-laden BATs are released from tension by cutting one anchor (see “tension release”), they rapidly degenerate within one day by shrinking

Uniaxial cyclic stretching on BATs was applied by sinusoidal elongation equating to 4% strain amplitude (0–8%) i.e. physiological load, or 8% (0–16%) i.e. overload [both regimes are defined according to [12, 16, 23]] for 2 h per day at 0.1 Hz (i.e. 720 cycles per day; Fig. S1) for a total of seven days. 0.1 Hz resembles the frequency used clinically in tendinopathy rehabilitation programs emphasizing slow, heavy loading [12]. Control BATs were unloaded, but were under tension between the two anchors of a plate (Fig. 1).

### Cell viability/cytotoxicity staining

BATs were washed three times in 3 ml Dulbecco’s phosphate-buffered saline (DPBS, D8537, Sigma-Aldrich, St. Louis, MO, USA) for 3 min at room temperature (RT) in the culture well. The LIVE/DEAD ™ Viability/Cytotoxicity Kit for mammalian cells (L3224, Invitrogen™, Carlsbad, CA, USA) was used according to manufactures introductions. As additional control, BATs were released from tension by cutting one anchor (“tension release”). Two µl ethidium homodimer-1 were mixed with 1 µl calcein AM in 1 ml DPBS and briefly vortexted. 100 µl were added to one BAT placed on a microscopy slide and cover slip. After 15 min incubation at RT in the dark, cellular signals were visualised via fluorescence microscopy (AxioPlan 2 imaging, Zeiss, Jena, Germany) using according filter sets: calcein in green indicating viable cells (ex/em 490 nm/515 nm); ethidium homodimer-1 in red indicating dead cells (ex/em 560 nm/630 nm).

### Gene expression analysis

Individual BATs were digested using collagenase P (1 mg/ml; 11213873001, Roche, Merck KGaA, Darmstadt, Germany) for 2 h at 37 °C. Cells were pelleted by centrifugation at 200 g for 5 min at RT, supernatant was removed and cells were lysed using buffer RLT Plus from the RNeasy® Plus Mini kit (74136, Qiagen, Hilden, Germany). For further RNA extraction the handbook’s protocol “Purification of Total RNA from Animal Cells” was followed. Synthesis of cDNA was carried out using qScript® cDNA SuperMix (95048-100, Quantabio, Beverly, MA, USA). Amplification reactions were set-up by mixing 5 µl 2xPerfeCTa® SYBR® Green SuperMix (95054-500, Quantabio) with 1 µl template cDNA (refers to 4 ng transcribed RNA) and primers (forward and reverse, final 10 µM each) with nuclease-free water (129114, Qiagen) in a total of 10 µl. Primers for the following genes were used: the tenogenic markers *Scx*, *Tnmd* and *Mkx*; the ECM-related genes *Col1a1* and *Col3a1* as well as the ECM-remodelling *Mmp3* (sequences see Table S1). Amplification was monitored in real-time on a Rotor-Gene Q (Qiagen) and quantified using *Glyceraldehyde 3-phosphate dehydrogenase* (*Gapdh*) as the housekeeping gene. Normalised gene expression was calculated using a primer efficiency corrected  equation [24] relative to the expression of unloaded control BATs.

### IL-6 cytokine quantification

Enzyme-linked immunosorbent assays (Elisa) targeting the proinflammatory cytokine IL-6 (Mouse IL-6 ELISA Kit, RAB0308, Sigma-Aldrich) were conducted using centrifuged (10,000 g for 10 min at 4^○^C) supernatant of cell culture medium from BATs. IL-6 concentration in 100 µl undiluted supernatant of individual BATs were measured in pg/ml. For quantification, values were normalised to unloaded controls.

### Hematoxylin and eosin staining

BATs were fixed in 4% paraformaldehyde/DPBS at 5 °C overnight in dark conditions followed by three washing steps in each 0.4% paraformaldehyde/DPBS and DPBS. Fixed BATs were embedded in paraffin blocks and sectioned into 5 μm thin longitudinal slices using rotary microtome RM2265 (Leica, Wetzlar, Germany). After dewaxing and rehydrating using graded alcohol, specimens were stained for 8 min in Mayers hemalum solution (109249, Merck) followed by rinsing with tap water for 10 min. Slides were immersed in 0.1% eosin G (C.I. 45380) solution (115935, Merck) for 5 min. Slices were dehydrated in an ascending alcohol and immersed two times in xylol for 3 min. Finally, coverslips were mounted on top of the slides after adding Histofluid (6900002, Marienfeld GmbH, Lauda-Königshofen, Germany). After drying overnight at RT, H&E stained specimens were scanned using NanoZoomer 2.0-HT (Hamamatsu Photonics, Shizuoka, Japan). The Fiji software tool “analyse particles” was used for image analysis [25] measuring nuclear roundness, area and cellular density.

### F-actin staining

Fixed BATs were used for visualisation of the cytoskeleton by F-actin staining. BATs were washed three times for 5 min by gentle agitation at RT in DPBS followed by incubation for 1 h at RT with 1x Phalloidin-iFluor 488 Reagent (ab176753, abcam, Cambridge, UK) in DPBS/1% BSA including nuclear counter-stain Hoechst 34580 (H21486, Invitrogen™) at final concentration of 1 µg/ml. After three wash steps in DPBS, BATs were placed on top of a slide and mounted with ProLong™ gold antifade (P36934, Invitrogen™) under a cover slip. Fluorescent cells in BATs were visualized by confocal laser scanning microscopy (LSM 710, Zeiss, Jena, Germany) using a 40×/1.2 C-Apochromat® (Zeiss) for magnification. Excitation was conducted through a 405 nm laser diode and a 488 nm argon laser (Zeiss). The systems spectral Quasar detector was set up to monitor specimens at 415–490 nm for Hoechst and 490–561 nm for Phalloidin-labelled probes. Maximum intensity projection of z-stacks was used and images were processed using the imaging software ZEN (Zeiss).

### Statistics and sample size

Multiple group comparisons (i.e. overload *versus* physiological load *versus* controls for gene expression or Elisa) were calculated by using Kruskal-Wallis test with Dunn’s multiple comparison followed by Bonferroni-Holm-Correction. Statistical differences between BATs subjected to overload *versus* physiological load with respect to nuclear roundness, area and density were additionally calculated using Mann-Whitney U-test. In all cases SPSS 26 (IBM) was used to calculate statistics. For samples sizes, see Table S2.

## Results

### Cell viability

**Fig. 2:**
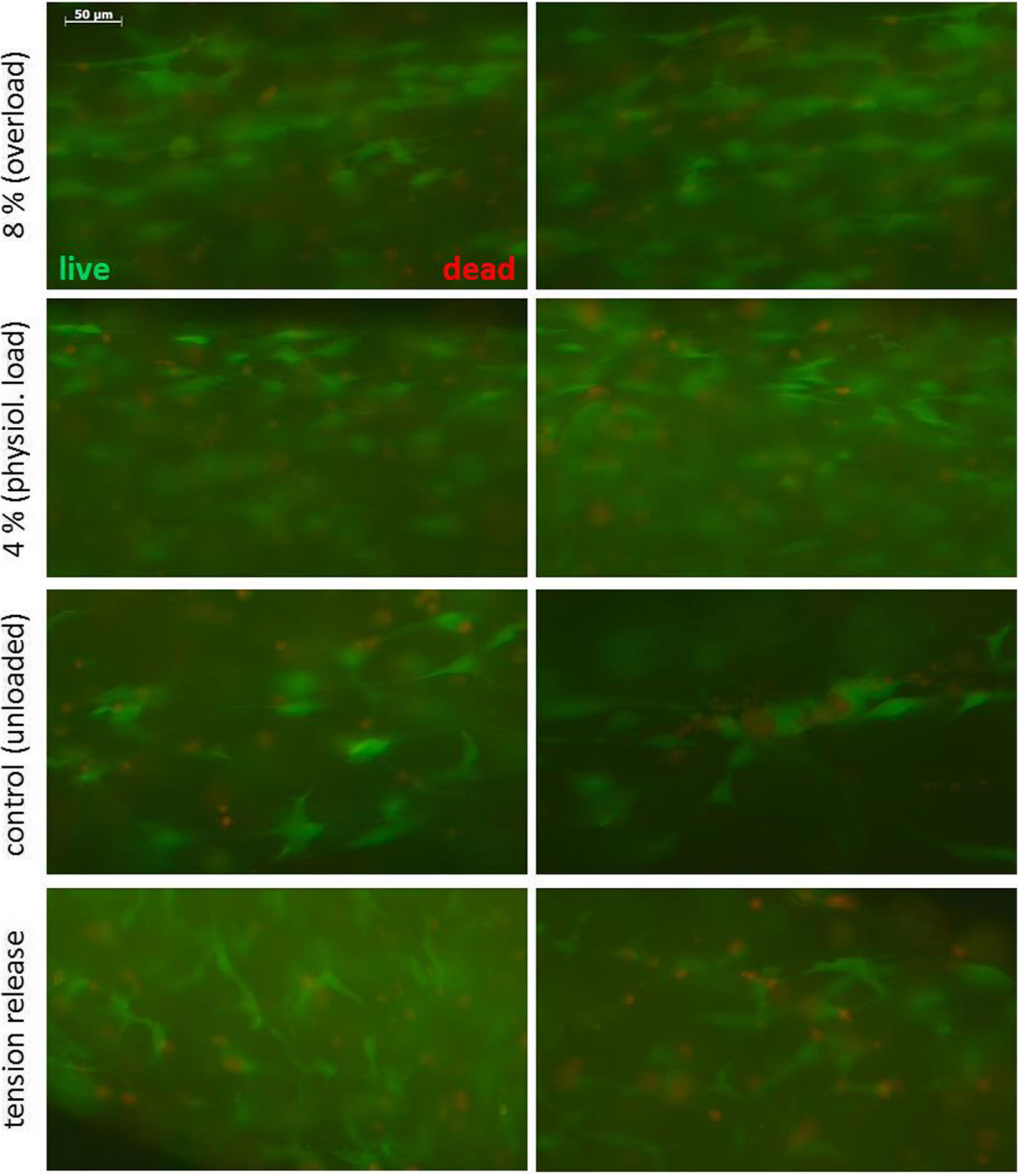
Cell survival in BATs after one week of maturation without load and another week with cyclic elongation at overload conditions (mean 8%) or physiological load (mean 4%) as well as unloaded controls and tension-released BATs. Three BATs of each treatment were stained with calcein AM (green fluorescent, viable cells) and ethidium homodimer-1 (red fluorescent, dead cells) to distinguish green live and red dead cells via fluorescence microscopy. There was no obvious difference in the ratio of live to dead cells among the treatments and most cells were alive. Only cells in tension-released BATs showed a lower ratio of live to dead cells compared to loaded or control BATs

The majority of cells in overloaded, physiologically loaded and unloaded control BATs were positive for calcein green fluorescent staining which indicates viable cells (Fig. 2). Few cells were positive for ethidium homodimer-1 red fluorescent staining indicating dead cells. However, there were no obvious differences in cell survival and viability between overloaded, physiologically loaded and unloaded BATs, i.e. most cells survived two weeks in BATs including one week maturation followed by another week under mechanical load. However, cells in tension-released BATs showed a lower ratio of live to dead cells compared to loaded or control BATs (Fig. 2).

### Gene expression

**Fig. 3:**
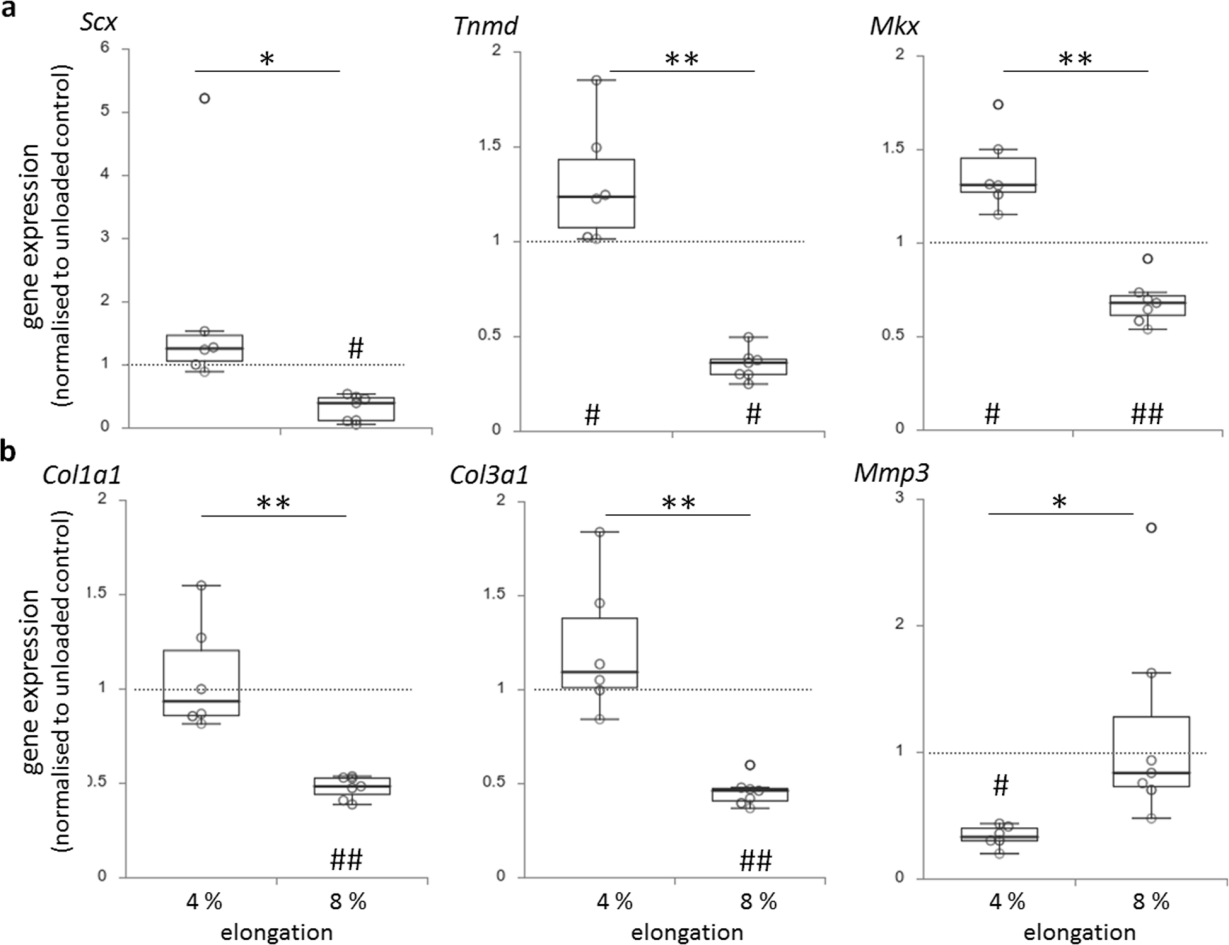
Expression of tenogenic (**a**) and ECM (**b**) related genes in BATs after physiological (4%) or overload (8%) elongation normalised to unloaded controls. Significant and highly significant differences between 4% *versus* 8% elongation are indicated by * (*p* ≤ 0.05) or ** (*p* ≤ 0.01) or by # (*p* ≤ 0.05) or ## (*p* ≤ 0.01) comparing loaded BATs *versus* unloaded control BATs. Dotted grey lines indicate mean expression of unloaded controls

Expression of tenogenic marker genes such as *Scx, Tnmd* and *Mkx* (Fig. 3A) as well as the ECM-related collagen genes *Col1a1* and *Col3a1* (Fig. 3B) were significantly decreased in BATs subjected to overload (ø 8% elongation) compared to physiological load (ø 4%) and unloaded controls (0%, yet under tension), respectively. *Mkx* and *Tnmd* were significantly increased in BATs subjected to physiological load compared to controls. In contrast, expression of *Mmp3* was significantly increased at overload compared to physiological load, but significantly decreased under physiological load compared to unloaded controls.

### IL-6 quantification

**Fig. 4:**
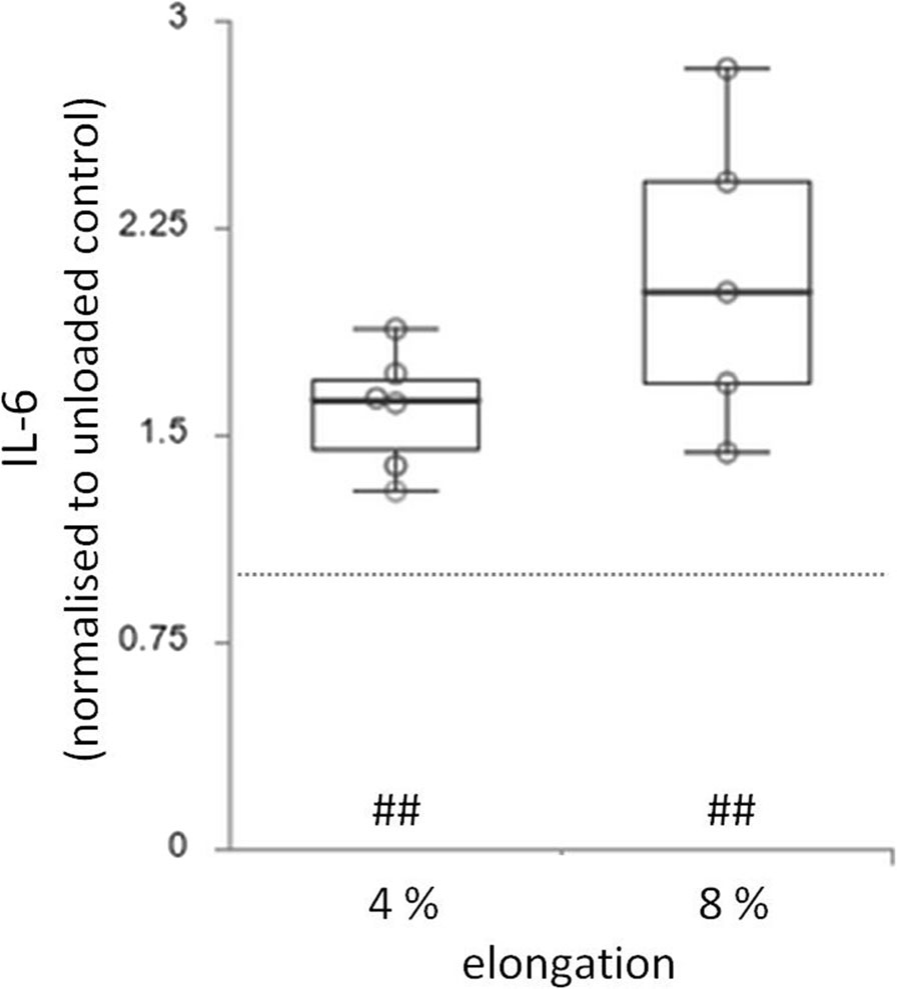
Quantification of interleukin-6 in cell culture supernatant of BATs subjected to physiological (4%) or overload (8%) elongation after normalisation to unloaded controls. Significant differences between loaded BATs *versus* unloaded controls are indicated by ## (*p* ≤ 0.01). Dotted grey line indicates mean IL-6 level of unloaded controls

Levels of IL-6 in culture supernatants of fibroblasts-laden BATs subjected to mechanical overload were similar (median 2.02; interquartile range 1.06) to BATs subjected physiological load (median 1.63; interquartile range 0.40) (Fig. 4). In both cases, IL-6 levels were significantly higher under overload or physiological load compared to unloaded controls (*p* = 0.004; *p* = 0.007).

### Histology

**Fig. 5:**
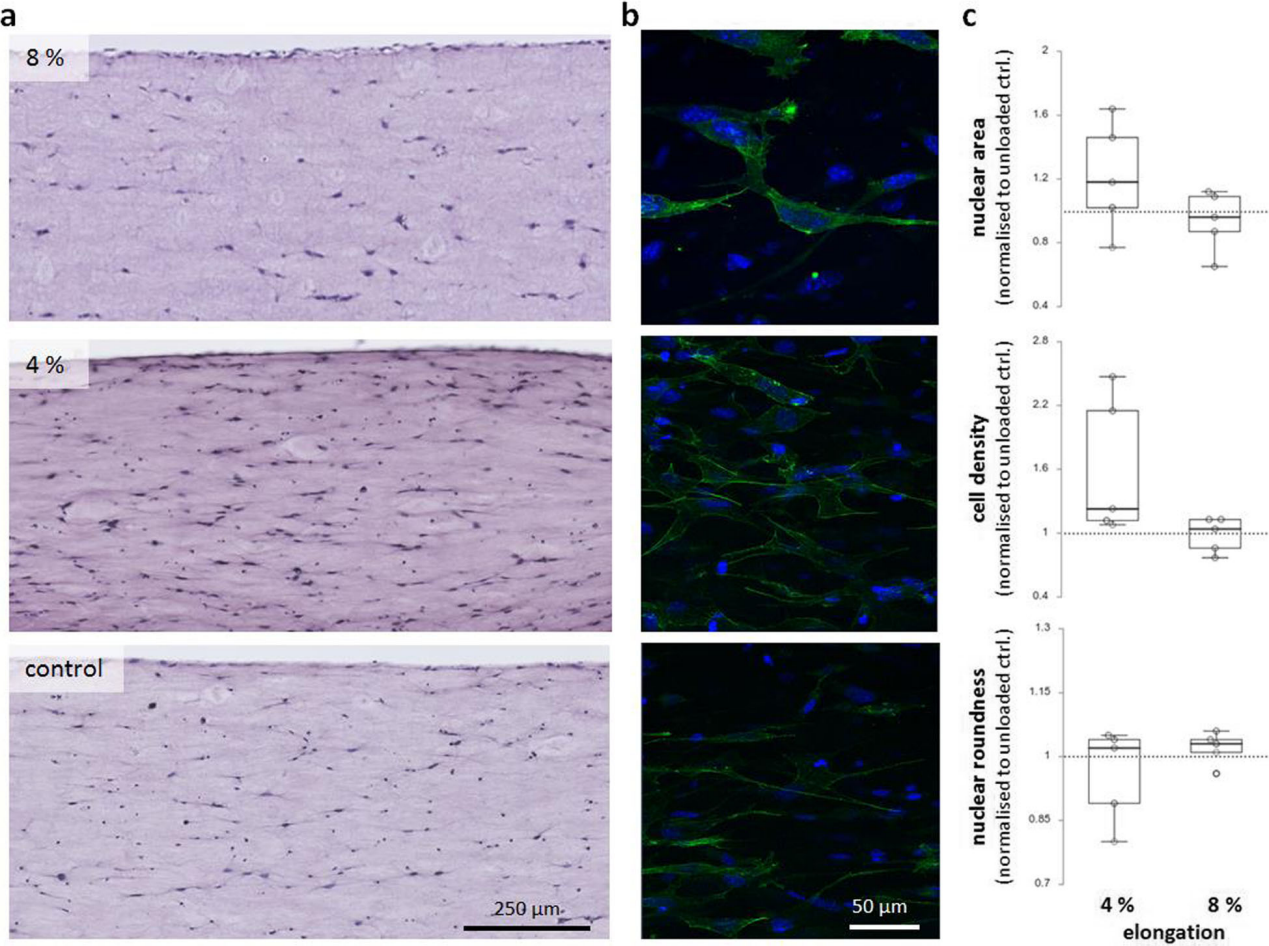
Histological and cellular analysis of BATs subjected to overload (8%) or physiological (4%) load elongation in comparison to unloaded controls. **a** Hematoxylin & eosin stain of longitudinal 5 μm slices. **b** Visualisation of the cytoskeleton in intact BATs by F-actin staining via Phalloidin (in green) and confocal laser scanning microscopy. Single z-stack images in 40x magnification are shown. Counterstaining was carried out using Hoechst 34580 staining dsDNA in the cell nucleus (blue). **c** Hematoxylin & eosin-stained micro slices were analysed for nuclear area, cell density and roundness (low values indicate an elongated shape). Dotted grey lines indicate mean value of unloaded controls

H&E staining of longitudinal sections of BATs showed a relative uniform distribution of C3H10T1/2 cells along the constructs (Fig. 5A). Longitudinal alignment of cells in strain direction with numerous cell-to-cell contacts was especially pronounced in BATs subjected to physiological load, whereas orientation of cells in control BATs appeared more randomly (Fig. 5A, B). Nuclear area (*p* = 0.222) and roundness of nuclei (*p* = 0.548) were not different between physiological and overload conditions (Fig. 5C). Cellular density did not differ significantly between overload and physiological load (*p* = 0.095) (Fig. 5C).

## Discussion

Various cell types embedded in 3D tendon-like constructs are able to respond to physiological load, mainly with enhanced expression of tendon-related markers, elongated cell shape and increased cellular alignment (reviewed in 1, 5, 6). This knowledge is confirmed in our study using fibroblasts-laden BATs. However, only few studies have analysed the impact of overload on 3D-cultered tenocytes or fibroblasts e.g. [26]. Using latter cell type seeded in BATs, here we show that mechanical overload decreases molecular and histological tendon-related markers, whereas physiological load increases tenogenesis.

Load, or at least tension, is essential for maturation and functioning of fibroblasts-laden BATs as shown in this study, since tension-released BATs quickly degenerated and shrank (Fig. 1). Similarly, 3D constructs using C3H10T1/2 fibroblasts showed decreased expression of the tendon-related genes *Scx, Tnmd* and *Col1a1* after tension release in comparison to constructs under tension [27]. Moreover, whereas cell-free BATs stayed loose and friable over time since they lack the ability of fibroblasts to remodel the matrix, cell-laden BATs acquired a thin, solid and tendon-like phenotype within one week when under tension (Fig. 1); this is consistent with findings from other studies using BATs [12, 28].

Survival of cells in 3D constructs over a longer time seems to require load or at least tension. Comparable cell survival was found for C3H10T1/2 cells after 15 days of culture or mechanical load [21]. This is similar to our findings showing that cell survival and viability did not differ among physiological, overload and unloaded controls (Fig. 2). Similarly, cyclic strain at different frequencies (0.3, 0.5, 1.0 Hz) and amplitudes (2, 4, 8%) subjected to Achilles tendon-derived stem cells (TDSCs) from rats in a 3D-bioreactor had no influence on their viability [29].

However, when BATs were subjected to overload (up to 16%) tenogenesis, i.e. expression of *Scx, Tnmd, Mkx, Col1a1* and *Col3a1*, was significantly decreased compared with a lower, physiological load (up to 8%) or unloaded controls (Fig. 3). Alike, Achilles TDSCs from rats that were cyclically stretched at 2%, 4% or 8% showed highest expression of *Col1a1*, *Tnmd* and *Scx* at intermediate 4% strain [29]. Interestingly, straining human mesenchymal stem cells cyclically at 10% or statically at 15% in 3D scaffolds resulted in upregulation of *Scx*, *Tnmd* and *Col1a1* [30, 31]. Moreover, the expression of *Mmp3*, a protein important for ECM remodelling, was increased in BATs subjected to overload compared to physiological load, while physiological load resulted in a reduced expression compared to static conditions (Fig. 3). Quantification of IL-6 in the supernatant revealed an increase due to load (both over and physiological) compared to static controls (Fig. 4). Using rodent TDSCs in 3D tendon-like constructs under proinflammatory stimulation (yet unloaded) resulted in increased expression of *Mmp3* and IL-6, whereas *Col1a1*, *Col3a1*, *Scx*, *Tnmd* and *Mkx* expression did not change compared to untreated controls [19]. However, as shown in our study, these genes responded to mechanical load, i.e. decreased by overload and increased by physiological load (Fig. 3). Further studies confirm that improper loading can induce expression of anabolic and catabolic genes. For example, rabbit Achilles tendons cultured *ex vivo* showed highest *Mmp3* expression at 3% (under-loaded), whereas *Col1a1* and *Col3a1* expression were highest at 6% or 9% strain (overload), respectively [3]. In parallel cell apoptosis and collagen disorientation occurred at 9% overload, whereas 6% strain maintained the structural integrity and cellular function best [3]. Similarly, loading of rat tenocytes at 15% induced cytoskeleton damage due to F-actin depolymerisation [32]. Thus, mechanical overload does not only induce gene expression changes, but can also lead to morphological changes. This is confirmed in our study, since fibroblasts in BATs subjected to overload had reduced nuclear area, probably due to a more rounded and less elongated shape compared to cells subjected to physiological load (Fig. 5). Even though the changes were not significant, they are similar to the changes seen in tendinopathic tissue. Longer stimulation periods might result in more pronounced alterations as tendinopathy is also a result of a longer alteration process.

Physiological load as used here increased tenogenic differentiation in comparison to mainly overload, but also to unloaded conditions (Fig. 3). This result confirms findings from other studies. For example, using fibroblasts-laden BATs strained with a comparable regime as ours (0.1 Hz for 2 h/d) at 5–10%, Scott et al. [12] showed an increased expression of *Scx* and *Col1a1* especially under cyclic compared to static load. Using the same fibroblasts in a 3D fibrinogen gel subjected to 10% cyclic load (1 h per day at 0.5 Hz, 15 d total), *Col1a1* expression was significantly increased [21]. Fibroblasts in BATs subjected to physiological load were also found to have minor changes in cellular morphology, e.g. an alignment of the cytoskeleton in strain direction (Fig. 5B, C). Other studies found in descriptive histological analysis that loaded fibroblasts or tenocytes have well-aligned cytoskeletal organization and elongated nuclei, whereas unloaded cells were more rounded and poorly differentiated [12, 21, 28, 33, 34]. Using quantitative analysis, we assessed comparable differences and alterations due to different loading conditions, even though the differences reached not significant values. Interestingly, cyclic overload at 15% but at higher frequency (1 Hz) as used in our study exerted a proinflammatory effect in rat Achilles tenocytes, mainly due to disruption of the cytoskeleton, which may also contribute to tendinopathy [32].

Future research about the impact of overload on (bioartificial) tendons should take into account that physiologic load of individual tendons differ among their function, age, sex, location and species [9]. Given the high worldwide prevalence of tendon injuries such as tendinopathies and the limited self-renewal capacity of tendons [35], it is of clinical importance to avoid potentially harmful overload movements that may occur during rehabilitation programs [36]. These challenges could be faced through an stepwise expansion of the current study by: (*i*) using BATs seeded with primary cells such as tenocytes or TDSCs preferably from humans to overcome potential limitations of a murine cell line [e.g. they are not preferentially committed to the tendon lineage as compared to TDSCs originating from native tissues, they may differentiate into other cells of the musculoskeletal system [22]], (*ii*) analysing the impact of further mechanostimulation variables such as frequency, duration or rest insertion in addition to amplitude, and (*iii*) straining *ex vivo* or genetically modified *in vivo* tendon models [37, 38] at overload.

## Conclusions

We mainly found that mechanical overload decreases gene expression of tendon-related markers and collagens, whereas expression of remodelling/inflammation markers increased. In contrast, physiological load increased expression of tendon-related markers in BATs and was accompanied by histological changes such as aligned cells in strain direction – an important marker also found in native tendon tissue [39]. Thus, our overload results show some potential parallels to tendinopathy, a tendon disease condition at which cell alignment and elongated morphology are lost and inflammatory and ECM remodelling marker genes are increased [40].

## Supplementary information

**Additional file 1**

## Data Availability

The datasets used and/or analysed during the current study are available from the corresponding author on reasonable request.
