# Supplementary material for: Mechanical overload decreases tenogenic differentiation compared to physiological load in bioartificial tendons
Source: J Biol Eng. 2022 Mar 3;16:5. doi: 10.1186/s13036-022-00283-y (PMC8896085; doi:10.1186/s13036-022-00283-y)
Supplement: Supplementary file 1 — Additional file 1 [file 13036_2022_283_MOESM1_ESM.docx]

**Supplementary Material**


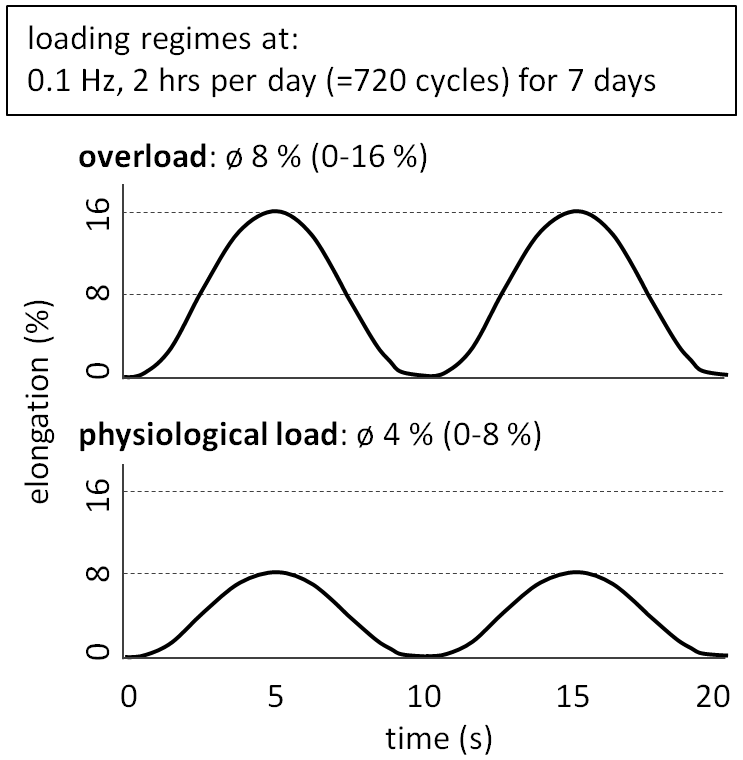


**Fig. S1** The two different loading regimes, i.e. overload and physiological load, used in this study. Both were used to stretch bioartificial tendons at 0.1 Hz for 2 h a day for a total of 7 days. Whereas overload load was on average 8% (ranging from 0-16%), physiological load was on average 4% (ranging from 0-8%).

**Table S1** Sequences of primers used for quantitative real-time PCR.

| **Gene** | **Forward sequence** | **Reverse sequence** |
| --- | --- | --- |
| *Mkx* | GGGAAAACAAGGCGAAGGAAC | CGGGGTGTCTGTGCGATAG |
| *Tnmd* | TGTACTGGATCAATCCCACTCT | GCTCATTCTGGTCAATCCCCT |
| *Scx* | Qiagen QuantiTect QT00166271 | |
| *Col1a1* | GGTCCACAAGGTTTCCAAGG | GTTCCAGGCAATCCACGAG |
| *Col3a1* | GCTGGAGTTGGAGGTGAAAA | GCAGCCTTGGTTAGGATCAA |
| *Mmp3* | TGGAGATGCTCACTTTGACG | ATGGAAACGGGACAAGTCTG |
| *Gapdh* | AGGTCGGTGTGAACGGATTT | TGAATTTGCCGTGAGTGGAG |

**Table S2** Samples sizes expressed as number of individual BATs used in the different experiments.

|  | 4% physiological load | 8% overload | unloaded control |
| --- | --- | --- | --- |
| Gene expression | 6 | 7 | 6 |
| Elisa | 6 | 5 | 6 |
| H&E stains | 5 | 5 | 5 |
| F-Actin stains | 3 | 3 | 3 |
